# Achiral Mannich-Base Curcumin Analogs Induce Unfolded Protein Response and Mitochondrial Membrane Depolarization in PANC-1 Cells

**DOI:** 10.3390/ijms18102105

**Published:** 2017-10-07

**Authors:** Gábor J. Szebeni, Árpád Balázs, Ildikó Madarász, Gábor Pócz, Ferhan Ayaydin, Iván Kanizsai, Roberta Fajka-Boja, Róbert Alföldi, László Hackler, László G. Puskás

**Affiliations:** 1 Avidin Ltd., Alsó kikötő sor 11/D, H-6726 Szeged, Hungary; g.szebeni@avidinbiotech.com (G.J.S.); a.balazs@avicorbiotech.com (Á.B.); i.madarasz@avicorbiotech.com (I.M.); pocz.gabor@hotmail.com (G.P.); i.kanizsai@avidinbiotech.com (I.K.); r.alfoldi@avidinbiotech.con (R.A.); hackler@avidinbiotech.com (L.H.); 2 Laboratory of Functional Genomics, Institute of Genetics, Biological Research Centre, Hungarian Academy of Sciences, Temesvári krt. 62, H-6726 Szeged, Hungary; 3 Cellular Imaging Laboratory, Institute of Plant Biology, Biological Research Centre, Hungarian Academy of Sciences, Temesvári krt. 62, H-6726 Szeged, Hungary; ayaydin.ferhan@brc.mta.hu; 4 Artificial Chromosome and Stem Cell Research Laboratory, Institute of Genetics, Biological Research Centre, Hungarian Academy of Sciences, Temesvári krt. 62, H-6726 Szeged, Hungary; fajka_boja.roberta@brc.mta.hu

**Keywords:** curcumin, endoplasmic reticulum stress, unfolded protein response, mitochondrial depolarization, caspase-3, apoptosis

## Abstract

Achiral Mannich-type curcumin analogs have been synthetized and assayed for their cytotoxic activity. The anti-proliferative and cytotoxic activity of curcuminoids has been tested on human non-small-cell lung carcinoma (A549), hepatocellular carcinoma (HepG2) and pancreatic cancer cell line (PANC-1). Based on the highest anti-proliferative activity nine drug candidates were further tested and proved to cause phosphatidylserine exposure as an early sign of apoptosis. Curcumin analogs with the highest apoptotic activity were selected for mechanistic studies in the most sensitive PANC-1 cells. Cytotoxic activity was accompanied by cytostatic effect since curcumin and analogs treatment led to G_0_/G_1_ cell cycle arrest. Moreover, cytotoxic effect could be also detected via the accumulation of curcuminoids in the endoplasmic reticulum (ER) and the up-regulation of ER stress-related unfolded protein response (UPR) genes: *HSPA5*, *ATF4, XBP1*, and *DDIT3*. The activated UPR induced mitochondrial membrane depolarization, caspase-3 activation and subsequent DNA breakdown in PANC-1 cells. Achiral curcumin analogs, C509, C521 and C524 possessed superior, 40-times more potent cytotoxic activity compared to natural dihydroxy-dimetoxycurcumin in PANC-1 cells.

## 1. Introduction

Turmeric spice obtained from the rhizomes of Curcuma longa has been used for centuries in traditional Chinese medicine and in Ayurveda in India based on its assumed anti-inflammatory, anti-microbial, and anti-cancer effects [1]. Curcuminoids make up 2–5% of turmeric powder and consist of dihidroxy-dimetoxycurcumin (curcumin, 77%), mono-demethoxycurcumin (17%) and bis-demethoxycurcumin (3%) [2]. Due to the empirical beneficial health effect of turmeric the consumption of curcumin as a dietary supplement increased worldwide. By today, curcumin has been designated “Generally Recognized as Safe (GRAS)” by the Food and Drug Administration of the United States [3]. The widespread consumption of turmeric and curcumin and its potential as a pharmaceutical laid down the basis for the need of proper scientific studies to reveal the effects of curcumin on human health as well as the design and synthesis of curcumin analogs which can be potentially introduced to the clinic. Our aim was to increase the cytotoxicity of Mannich-type curcumin analogs and reveal their mechanism of action in a cellular system.

Tumorigenesis is a multistep process in which curcumin has been implicated to suppress the growth of tumor cells by reducing their proliferation and survival as well as to induce tumor cell death [2,4,5]. It has been published that curcumin inhibited the proliferation and induced the apoptosis of non-small-cell lung carcinoma cells (A549 cell line) by the up-regulation of microRNA-192-5p and suppression of the PI3K3/Akt signaling pathway [6]. In another study apoptosis of A549 cells induced by curcumin relied on oxidative stress and Mitogen-activated protein kinase (MAPK) pathway [7]. Curcumin attenuate not only tumor cell viability but also the metastatic ability of A549 cells by blocking the adiponectin receptor 1 [8] and glucose transporter 1 (GLUT1) [9]. Therefore, we tested our novel curcumin analogs on the non-small cell lung cancer (NSCLC) cell line, A549.

Curcumin caused apoptosis of human hepatocellular carcinoma HepG2 cells as well, via disruption of mitochondrial membrane potential [10] and counteracted fatty acid synthase [11]. In our study, we tested our analogs on both A549 cells as well as on the hepatocellular carcinoma (HCC) cell line HepG2.

Finally, we focused on PANC-1 cells, because among solid tumors, pancreatic malignancy has the highest mortality rate with the overall 5-year survival being less than 6% [12]. The possible application of curcumin in pancreatic cancer has been reported earlier where the authors showed that PANC-1 cells were sensitive to curcumin treatment via reduction of the inhibitors of apoptosis [13] and through the induction of forkhead box O1 [14].

Here, we present achiral Mannich-type curcumin analogs with potential cytotoxic activity on human NSCLC, hepatocellular carcinoma, and pancreatic cancer cell lines.

## 2. Results and Discussion

### 2.1. Achiral Curcumin Analogs Impaired Tumor Cell Viability

**Figure 1:**
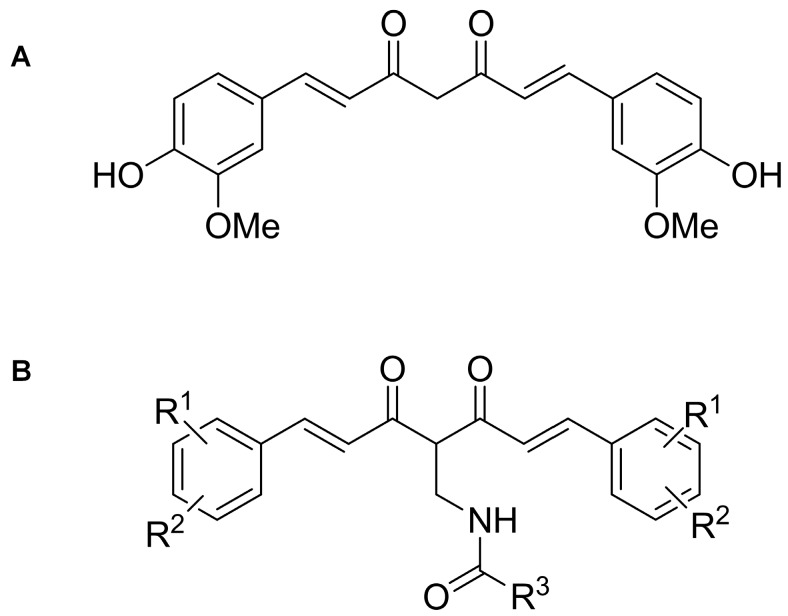
Curcumin and the general structure of achiral Mannich-type curcumin analogs. R^1^, R^2^ = F, OH, OMe, COOH, R^3^ is substituted by aryl or alky groups (each of the substitutes are specified in Figure S1.).

Recently, we have reported the potent cytotoxic activity of racemic Mannich-type curcumin analogs [15,16,17]. Here, we report the cytotoxic activity of the achiral curcumin analog library (C500 series) (Figure 1 and Figure S1).

The substitution pattern was carefully chosen based on the already established structure-activity relationship (SAR) results [17], with the ultimate aim to design a simpler and achiral library of curcuminoids with an improved pharmacokinetic profile. All intermediates and compounds were synthesized based on the previously established method [17,18]. As a pre-screening we tested the anti-proliferative effect of the first analog, C501 by measuring the confluency by live cell analyzer of C501 treated A549 cells (Figure S2). Since the anti-proliferative activity of C501 reached its maximum effect at 3.125 µM, we used lower concentrations of the members of C500 series (0.16–5 µM) in the subsequent experiments. We defined the dose-response curves of the 23 members of C500 series by the significantly more sensitive resazurin assay on A549, HepG2, and PANC-1 cells (Figure S3). The half maximal inhibitory concentration (IC_50_) values are presented in Table 1. The IC_50_ value of curcumin was above the pharmacologically favorable range 27.11 µM, 14.53 µM, and 30.57 µM in A549, HepG2, and PANC-1 cells, respectively. Six molecules were completely inactive at the applied concentrations (C514, C520, C525, C526, C529, and C530) and were not tested further. This was in full accordance with previously established SAR results [17,18], that the presence of functionalities such as carboxylic acid, dihydroxyphenyl, or *para*-hydroxy substituents resulted in the complete loss of cytotoxic activity. Compounds which possessed low anti-proliferative activity (IC_50_ > 5 µM) were also excluded from further testing (C502, C510, C513, C516, C517, C519, C532, and C533). The current project focused on curcumin analogs with potent cytotoxic activity on three different cell lines: A549, HepG2, and PANC-1. Nine of the 23 novel curcumin analogs (C501, C503, C504, C505, C509, C515, C518, C521, and C524) hampered the viability of all three tested cell lines when used below 5 µM.

### 2.2. Curcumin Analogs Induced Phosphatidylserine Exposure of A549, HepG2 and PANC-1 Cells

**Figure 2:**
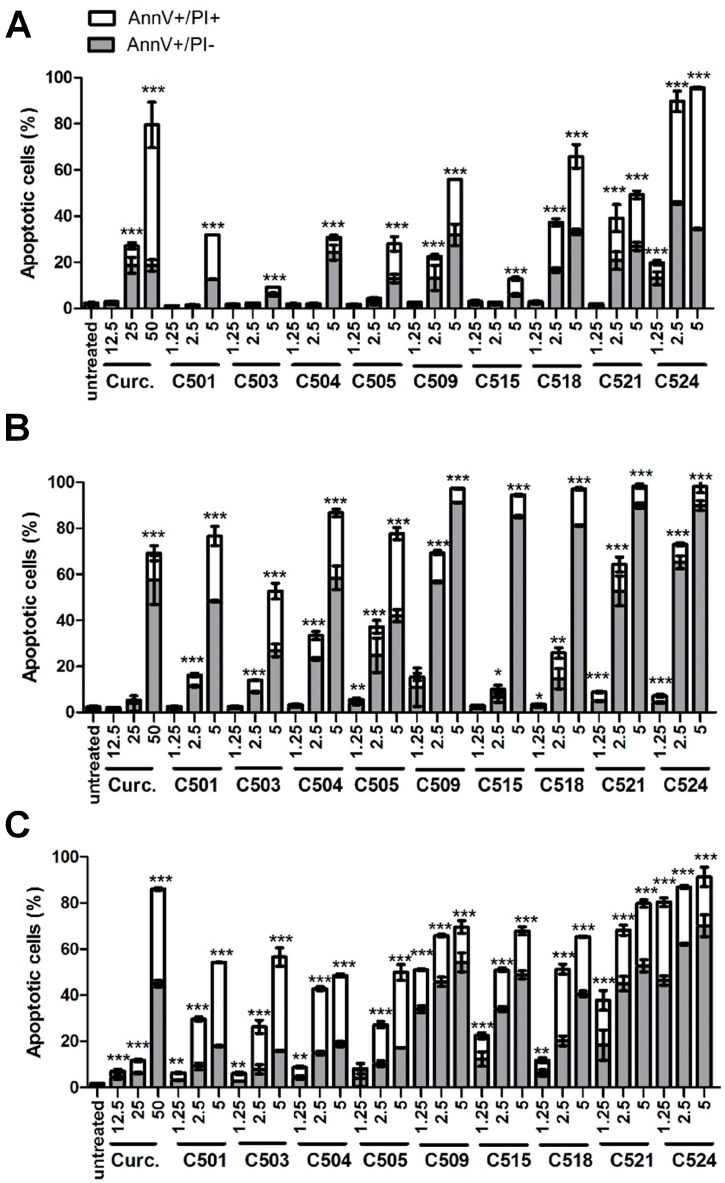
Curcumin and analogs induced phosphatidylserine exposure on tumor cells. A549 (**A**), HepG2 (**B**), and PANC-1 (**C**) cells were incubated with curcumin (Curc.) and curcumin analogs with the indicated concentrations (μM) as described in Section 3.3.1. Materials and Methods. The results are shown as arithmetic mean values of the summary of early (AnnV^+^/PI^−^, gray column) and late apoptosis (AnnV^+^/PI^+^, white column) of two samples ±SD, * *p* < 0.05, ** *p* < 0.01, *** *p* < 0.001.

In order to clarify whether the viability of A549, HepG2, and PANC-1 cells was hampered by apoptosis or necrosis, we carried out annexin V-propidium iodide (PI) staining on the cells treated by the nine selected candidates. Curcumin and its analogs showed dose dependent phosphatidylserine exposure that suggests potent apoptotic activity (AnnV^+^/PI⁻ early apoptosis and AnnV^+^/PI^+^ late apoptosis) in A549 (Figure 2A), HepG2 (Figure 2B), and PANC-1 cells (Figure 2C) without the appearance of a massive, only PI positive necrotic population (Figure S4).

In order to identify the most active analogs, we summarized the percentage of total apoptotic cell populations at the lowest applied concentration for each analog in Table 2. Three compounds stood out with appreciable activity, C509, C521, and C524, therefore only these were included in further experiments. All three molecules share the common C-4 chloroacetamidomethyl and either the *meta*-hydroxy or methoxy substituents on their side-chains. An analog with moderate activity, was also included (C501) in our analyses. Since PANC-1 cells were the most sensitive in our study, additionally the 5-year overall survival has been reported to be the lowest for pancreatic cancer (less than 6%) among other solid malignancies [12], we used PANC-1 cells for subsequent mechanistic studies to reveal how novel curcumin analogs counteract cancer cell viability.

### 2.3. Curcumin Analogs Caused G_0_/G_1_ Cell-Cycle Arrest of PANC-1 Cells

**Figure 3:**
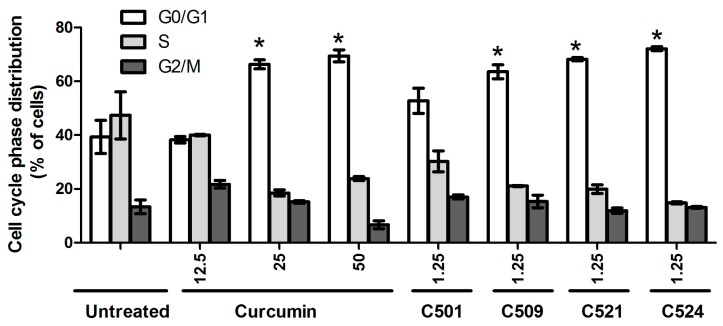
Curcumin and analogs caused G_0_/G_1_ arrest of PANC-1 cells. Cells were treated with curcumin and curcumin analogs with the indicated concentrations (μM) in the figure and G_0_/G_1_, S, G_2_/M cell cycle phase distributions were analyzed by flow cytometry as described in Section 3.3.2. Materials and Methods. The results are shown as arithmetic mean values of two samples ±SD, * *p* < 0.05.

In addition to cytotoxicity, we also investigated the cytostatic effect of our curcumin analogs on PANC-1 cells. Curcumin (25 µM), and C509, C521, and C524 (both 1.25 µM) significantly increased G_0_/G_1_ population of PANC-1 cells (up to 80%) compared to the untreated control cells (40%) (Figure 3 and Figure S5).

According to several reports it is unequivocal that curcumin can dysregulate the cell cycle. It has been reported that natural curcumin caused G_2_/M mitotic catastrophy, in relation to the G_2_/M phase in head and neck squamous cell carcinoma or in bovine aortic endothelial cells after 24 h incubation [19,20], it was also shown that curcumin caused not only G_1_/S but also G_0_/G_1_ cell cycle arrest in human prostate cancer cells [21,22]. The differences in cell cycle dysregulation may be cell type specific or may depend on the different experimental conditions, incubation time period, the concentration and formulation of curcumin.

### 2.4. Curcumin Analogs Induced ER (Endoplasmic Reticulum) Stress and Mitochondrial Membrane Depolarization

**Figure 4:**
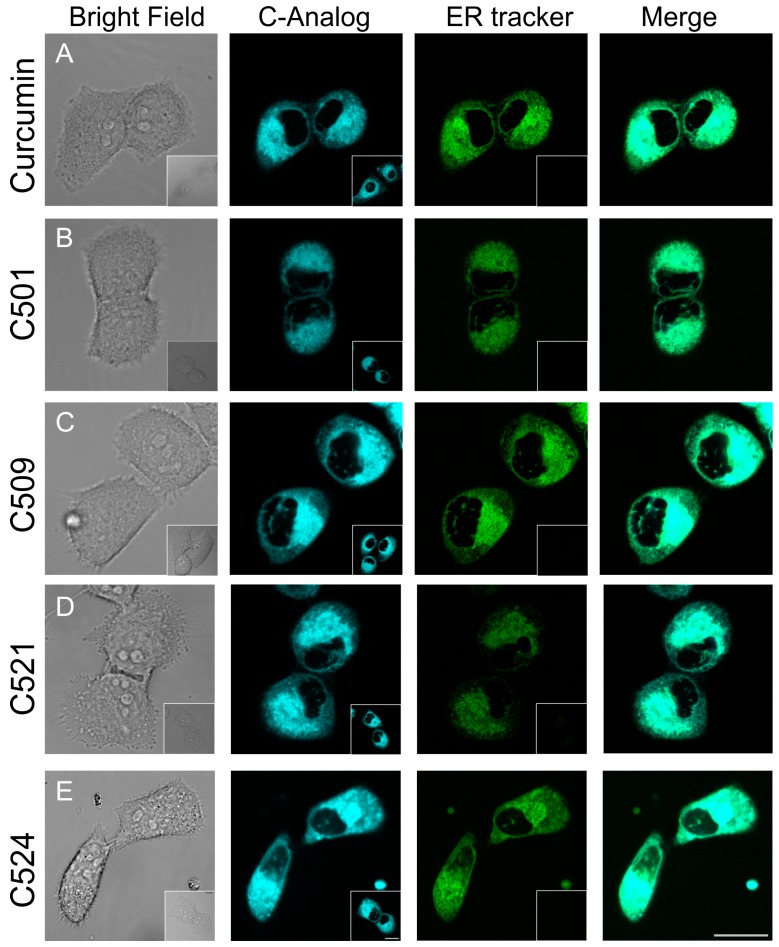
Curcumin and analogs localized in the endoplasmic reticulum of PANC-1 cells. Cells were treated with curcumin and analogs (**A**–**E**), 1 µM and 5 min. The subcellular localization of curcumin and its analogs was assessed with endoplasmic reticulum (ER) tracker co-localization by laser scanning confocal microscopy. To further minimize channel crosstalk, only curcumin analog labeled samples were also prepared (inset images) and used as reference for image capturing conditions for curcumin-ER tracker dual labelled samples. Representative images are shown. Scale bar at C524 inset image is valid for all insets (controls). Scale bar at the lower right corner of C524 is valid for all ER co-localization images. Both scale bars are 20 µm.

Among others, we have previously shown that perturbing the homeostasis of the ER could reduce cellular viability [23]. It was recently published that natural curcumin caused ER stress mediated apoptosis in cervical cancer cells [24] and in A549 cells [7]. In order to clarify whether the apoptotic effect of curcumin analogs in PANC-1 cells relied on the ER stress related mitochondrial apoptotic pathway, we followed the accumulation of the tested agents. Since curcumin possesses inherent fluorescence [25] and our analogs retained this property, we tested the subcellular localization of curcumin and our analogs in PANC-1 cells by fluorescence confocal microscopy. Similarly to curcumin, our analogs localized in the ER which was further verified by ER tracker co-localization (Figure 4).

The subcellular localization of curcumin analogs in the ER may activate an adaptive response to ER stress known as the unfolded protein response (UPR), which up-regulates ER chaperons, halts translation of secretory proteins, and degrades misfolded proteins. If the ER stress is irreversible the UPR mediates apoptotic cell death to restore the cell homeostasis [26]. ER stress was monitored by the induction of UPR related genes: *HSPA5* (Heat Shock Protein Family A (Hsp70) Member 5), *ATF4* (Activating Transcription Factor 4), *XBP1* (X-Box Binding Protein 1), and *DDIT3* (DNA Damage Inducible Transcript 3)*.* HSPA5 is a chaperon, master regulator of the UPR [27], ATF4, and XBP1 are transcriptional activators of UPR genes and chaperons [28], DDIT3 (encoding CHOP) is a transcription factor mediating ER stress related apoptosis [17,26,29].

**Figure 5:**
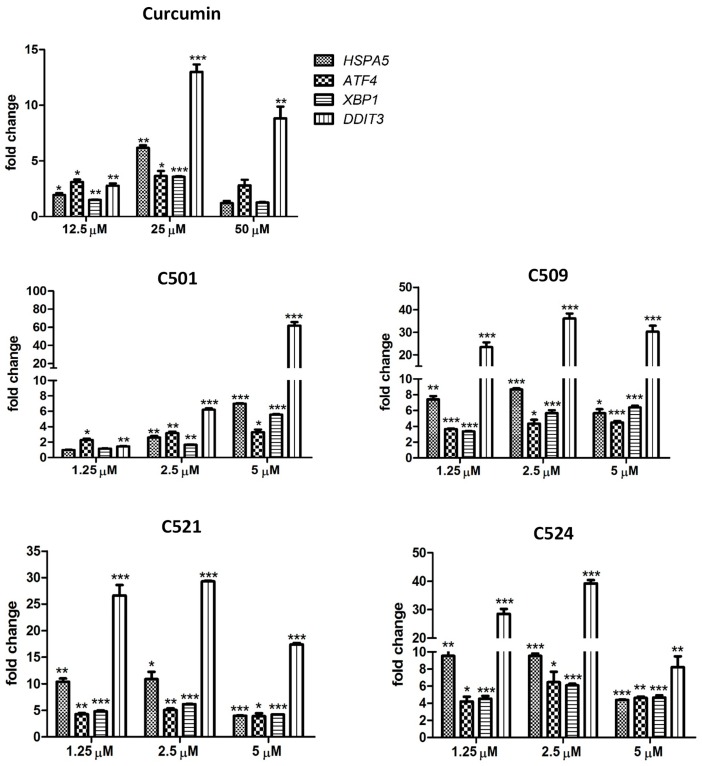
Curcumin analogs induced unfolded protein response during ER stress. Gene expression of unfolded protein response genes: *HSPA5, ATF4, XBP1, DDIT3* (arithmetic means of fold changes ±SD) in PANC-1 cells treated with curcumin and curcumin analogs after 12 h. Gene expression was analyzed by qRT-PCR (quantitative real-time polymerase chain reaction) as described in the Section 3.5. Materials and Methods, * *p* < 0.05, ** *p* < 0.01, *** *p* < 0.001.

Curcumin and the tested analogs induced the expression of all tested genes 12 h after treatment (Figure 5). The up-regulation of *HSPA5* was two-fold by 12.5 μM curcumin and around 10-fold by 1.25 μM C509, C521, and C524 treatment. *ATF4* and *XBP1* were around 4–5-fold up-regulated upon curcumin and curcumin analog stimulation. The highest increase was detected in case of *DDIT3*, where a 13-fold increase was detected with curcumin (25 μM), while the analogs induced around 30-fold overexpression at 1.25 μM, only C501 showed 60-fold change at 5 μM. *DDIT3* overexpression may suggest that in curcumin and curcumin analog treated cells the UPR progressed to a state where homeostasis cannot be restored and the cells are committed to an apoptotic fate [29,30]. The concentration dependent decline of gene expression changes (50 μM curcumin and 5 μM C509, C521, and C524) may be due to the advanced apoptotic program.

**Figure 6:**
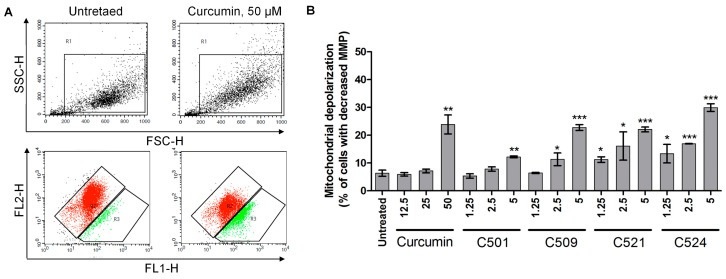
Curcumin and analogs depolarized mitochondria of PANC-1 cells examined by JC-1 staining. (**A**) Representative SSC-FSC and FL2-FL1 dot plots and (**B**) arithmetic means of percentages (±SD) of cells with decreased MMP at 24 h following treatment (µM). JC-1 was analyzed by flow cytometry as described in Section 3.3.4. Materials and Methods, * *p* < 0.05, ** *p* < 0.01, *** *p* < 0.001.

The perturbation of the homeostasis of the ER by curcumin analogs may confound the subcellular oxidative homeostasis affecting mitochondrial membrane potential (MMP) [31]. Therefore, mitochondrial membrane depolarization was examined by JC-1 (5,5′,6,6′-tetrachloro-1,1′,3,3′-tetraethylbenzimidazolocarbocyanine iodide) staining following curcumin and curcumin analog treatment of PANC-1 cells. Not only curcumin but also our analogs depolarized mitochondria of PANC-1 cells in a concentration dependent manner (Figure 6). The effect of curcumin on MMP was significant at 50 µM, while C501 analog was effective at 5 µM, C509 at 2.5 µM, and C521 and C524 at 1.25 µM.

### 2.5. Curcumin Analogs Induced Caspase-3 Activation and DNA Fragmentation

**Figure 7:**
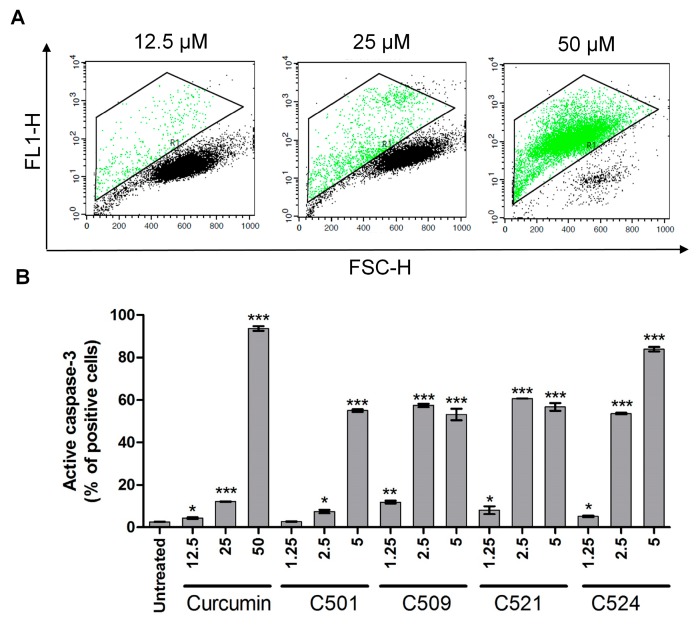
Curcumin and analogs activated caspase-3 in PANC-1 cells. (**A**) Representative FL1-FSC dot plots and (**B**) arithmetic means of percentages ±SD of cells with active caspase-3 show data of cells treated with curcumin and curcumin analogs with the indicated concentrations (µM) on the graph for 72 h. Active caspase-3 was analyzed by flow cytometry as described in Section 3.3.3. Materials and Methods, * *p* < 0.05, ** *p* < 0.01, *** *p* < 0.001.

Mitochondrial membrane depolarization may lead to the activation of caspases which renders cells for apoptosis [32]. Effector caspase-3 activation occurred in PANC-1 cells following curcumin and curcumin analog treatment in a concentration dependent manner (Figure 7). Among curcumin analogs C524 showed the highest induction of caspase-3 at 5 µM. 

**Figure 8:**
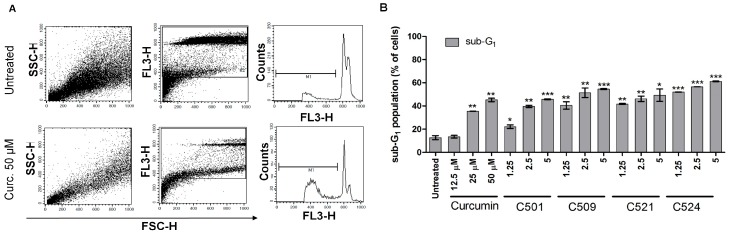
Curcumin analogs caused DNA breakdown of PANC-1 cells. (**A**) Representative SSC-FSC, FL3-FSC dot plots (gating out debris) and FL3 histograms are presented. (**B**) The arithmetic mean values of replicates ±SD are presented. Cells were treated with curcumin and curcumin analogs with the indicated concentrations (µM) in the figure in duplicates for 72. Sub-G1 population was analyzed by flow cytometry as described in Section 3.3.2. Materials and Methods, * *p* < 0.05, ** *p* < 0.01, *** *p* < 0.001.

Apoptosis executed by the activation of caspases results in the internucleosomal degradation of genomic DNA which can be studied by flow cytometry as a hypodiploid sub-G_1_ fraction of the cell population [33]. In order to confirm that event we analyzed the percentage of cells with degraded DNA content (Figure 8A). Using PANC-1 cells, our results showed that treatment with curcumin resulted in DNA degradation at 25 µM, while curcumin analogs exerted a significant effect at 1.25 µM. Cytotoxicity of the curcumin analogs resulted in DNA breakdown as the final step of apoptosis in a concentration dependent manner (Figure 8B).

## 3. Materials and Methods

### 3.1. Cell Culturing and Treatments

Cells were purchased from the American Type Culture Collection (ATCC, Manassas, Virginia, USA). A549, HepG2 cells were maintained in Dulbecco’s Modified Eagle Medium/Nutrient Mixture F-12 (DMEM/F12) 10% fetal calf serum (FCS, Gibco) and PANC-1 cells were maintained in Roswell Park Memorial Institute 1640 medium (RPMI-1640) 10% FCS, pH of the cell culture media was controlled to be between 7.2–7.4 prior use. Media were supplemented with 2 mM GlutaMAX, and 100 U/mL penicillin, 100 µg/mL streptomycin (Life Technologies, Carlsbad, California, USA). The cell cultures were maintained at 37 °C in a humidified incubator in an atmosphere of 5% CO_2_ (Sanyo, Japan).

Curcumin and curcumin analogs were dissolved in dimethyl sulfoxide (DMSO) at 10 mM concentration. Since DMSO can be toxic for cellular systems, the stock solution was further diluted in serial dilutions in all cases in the appropriate cell culture media. The intermediate dilution of curcumin was 250 µM (40× dilution), it was serially diluted to 125 µM (80× dilution) and it was further diluted to 62.5 µM (160× dilution), then each intermediate dilution was further diluted 5× when it was added to the cells, so the treatments of curcumin were 50 µM (200×), 25 µM (400×) and 12.5 µM (800×). The intermediate dilution of curcumin analogs was 25 µM (400×), it was serially diluted to 12.5 µM (800× dilution) and it was further diluted to 6.25 µM (1600× dilution), then each intermediate dilution was further diluted 5× when it was added to the cells so the treatments of curcumin analogs were 5 µM (2000×), 2.5 µM (4000×), and 1.25 µM (8000×).

### 3.2. Resazurin Viability Assay

The viability of A549, HepG2, and PANC-1 cells was determined by the fluorescent resazurin assay as described previously [34]. Briefly, cells (6 × 10^3^) were seeded into 96-well plates (Corning Life Sciences, Tewksbury, MA, USA) in media. Cells were cultured overnight before treatment. Viability was determined after a 72 h incubation in treated and non-treated cells. Resazurin reagent (Sigma-Aldrich) was dissolved in phosphate buffered saline (PBS, pH 7.4) at 0.15 mg/mL concentration, sterile filtered (0.22 µm, Merck Millipore) and aliquoted at −20 °C. Samples were treated in 120 µL volume with a final concentration of 25 µg/mL resazurin. After 2 hours incubation at 37 °C 5% CO_2_, fluorescence (530 nm excitation/580 nm emission) was recorded on a multimode microplate reader (Cytofluor4000, PerSeptive Biosytems, Framingham, MA, USA). Viability was calculated in relation to untreated control cells and blank wells containing media without cells. The presented IC_50_ values (half maximal inhibitory concentration) were determined based on dose-response curves plotted in GraphPad Prism^®^ 5 (La Jolla, CA, USA).

### 3.3. Flow Cytometry

#### 3.3.1. Detection of Phosphatidylserine Exposure

Cells (5 × 10^4^) were plated in 24-well tissue culture plates (Corning Life Sciences) and treated in 500 μL volume with increasing concentrations of test compounds in duplicates. After 72 h the supernatants were harvested. Cells were washed with PBS, trypsinized, pooled with the corresponding supernatant and pelleted by centrifugation (2000 rpm, 5 min). The pellet was resuspended in annexin V binding buffer (0.01 M HEPES (4-(2-hydroxyethyl)-1-piperazineethanesulfonic acid), 0.14 M NaCl and 2.5 mM CaCl_2_). Annexin V-Alexa Fluor^®^ 488 (AnnV, Life Technologies, 2.5:100) was added to the cells, which were then kept in the dark at room temperature for 15 min. Propidium iodide (PI, Sigma-Aldrich, St. Louis, MO, USA) was added at a final concentration of 10 μg/mL right before the acquisition of samples. Cells (2 × 10^4^ events) were analyzed on a FACSCalibur flow cytometer using CellQuest software (Becton Dickinson, Franklin Lakes, NJ, USA). The percentage of annexin V-Alexa Fluor^®^ 488 positive (FL1 channel, 530/30 nm filter) and propidium iodide negative (FL3 channel, 670 nm filter) early apoptotic cells and FL1 positive and FL3 positive late apoptotic cells were determined. The total apoptotic population includes both early and late apoptotic cells. Bar graphs were created by GraphPad Prism^®^ 5.

#### 3.3.2. Cell Cycle and Sub-G_1_ Analysis

PANC-1 cells (5 × 10^4^) were plated in 24-well tissue culture plates (Corning Life Sciences) in RPMI 10% FCS and were treated in 500 µL media containing test compounds in duplicates. After 72 h the supernatant was harvested. Cells were washed with PBS, trypsinized, pooled with the corresponding supernatant and centrifuged (2000 rpm, 5 min). Pellet was resuspended in DNA binding buffer (1× PBS, 0.1% tri-sodium-citrate, 10 µg/mL PI, 0.1% Triton X-100, 10 µg/mL RNase A, (Sigma-Aldrich)). After incubation at room temperature for 30 min 2 × 10^4^ events were acquired on a FACSCalibur flow cytometer (Becton Dickinson). Sub-G_1_ apoptotic population was analyzed on FL3 histograms using CellQuest^TM^ software (Becton Dickinson). We gated out doublets for cell cycle analysis which was based on FL2-A/FL2-W dot plots, using Modfit software (Becton Dickinson). Bar graphs were created by GraphPad Prism^®^ 5.

#### 3.3.3. Immunofluorescence

PANC-1 cells (1 × 10^5^) were plated in 24-well tissue culture plates (Corning Life Sciences) in RPMI 10% FCS and were treated in 500 µL media containing test compounds. After 72 h the supernatant was harvested. Cells were washed with PBS, trypsinized, pooled with the corresponding supernatant and centrifuged (2000 rpm, 5 min). Pellet was resuspended and fixed in 3.5% PBS buffered formaldehyde (Molar Chemicals) for 10 min. Cells were washed with FACS-buffer (2% FCS in PBS), centrifuged (2000 rpm, 5 min). Cells were permeabilized in permeabilization buffer (1% FCS, 0.1% saponin (Sigma-Aldrich) in PBS pH 7.4) for 5 min. Cells were washed with FACS buffer (2% FCS in PBS), centrifuged (2000 rpm, 5 min). Rabbit polyclonal anti-cleaved caspase-3 antibody (9661S, Cell Signaling) was added at 1:600 dilution in FACS buffer. After incubation for 1 h at 4 °C samples were washed two times with FACS buffer. The secondary antibody, polyclonal goat anti-rabbit IgG conjugated with Alexa Fluor^®^ 488 (A11008, Thermo Fisher Scientific, Waltham, MA, USA) was diluted to 1:600 and incubated with the cells for 30 min at 4 °C. After washing, 300 µL FACS buffer was added for acquisition with the FACSCalibur flow cytometer acquiring 2 × 10^4^ events at FL1 channel. Data were analyzed using CellQuest^TM^ software (Becton Dickinson) gating out debris. In order to calculate the signal to noise ratio, median fluorescence intensity (MFI) was calculated by the following equation: MFI = 10^[(Median stained − Median unstained, untreated)/Chd]^, where Chd (the number of channels per decade) equals 256, [35,36]. Bar graphs were created by GraphPad Prism^®^ 5.

#### 3.3.4. Detection of the Loss of Mitochondrial Membrane Potential

PANC-1 cells (1 × 10^5^) were plated in 24-well tissue culture plates (Corning Life Sciences) in RPMI 10% FCS and were treated in 500 µL media containing test compounds. After 24 h, the supernatants were harvested. Cells were washed with PBS, trypsinized, pooled with the corresponding supernatant and centrifuged (2000 rpm, 5 min). Pellet was resuspended and incubated for 15 min in 5 µg/mL JC-1 (5,5′,6,6′-tetrachloro-1,1′,3,3′-tetraethylbenzimidazolocarbocyanine iodide, Chemodex) containing media in final volume 300 µL at 37 °C. Finally, using FL2 (585/42 nm)-FL1 (530/30 nm) channels, the red–green fluorescence intensity of 1 × 10^4^ events was acquired immediately on FACSCalibur flow cytometer. Data were analyzed using CellQuest^TM^ software (Becton Dickinson) gating out debris. Bar graphs were created by GraphPad Prism^®^ 5.

### 3.4. Confocal Laser Scanning Microscopy

PANC-1 cells (2 × 10^5^) were plated on µ-Slide 8 Well Glass Bottom dishes (Ibidi^®^,Martinsried, Germany) in 500 µL media and placed in a humidified incubator at 37° C 5% CO_2_. After 24 h culturing, detection of curcumin, curcumin analogs (1 µM, 5 min) and ER tracker green (1 µM, 5 min, Thermo Fisher) were performed using Olympus Fluoview FV1000 confocal laser scanning microscope (Olympus Life Science Europa GmbH, Hamburg, Germany ) in triplicates. Microscope configuration was the following: objective: UPLFLN 40× (oil, NA:1.3); dichroic mirror: 20/80, excitation: 405 nm (curcumin analogs), 514 nm (ER tracker); detection range: 425–475 nm (curcumin), 530–630 nm (ER tracker); scanning dimensions: 512 × 512 pixel; sampling speed: 2 µs/pixel; confocal aperture: 182 µm; zoom: 5×; line averaging: 2×. Curcumin analogs and ER tracker green images were pseudocolored as cyan and green, respectively. Transmitted light images were also captured with 514 nm laser and paired with each fluorescence image. ER tracker green imaging was performed using 514 nm laser instead of 488 nm laser to minimize channel crosstalk. Composite images were prepared using CorelDraw^®^ Graphics Suite X7 (Corel, Ottawa, Canada).

### 3.5. Gene Expression Analysis

PANC-1 cells (2 × 10^6^) were plated in 60 mm tissue culture dishes (Corning Life Sciences) in RPMI 10% FCS. Twenty-four hours later cells were treated in 5 mL volume with test compounds. After 12 h treatment, nucleic acid preparation was done by using the Bioneer RNA purification kit (Bioneer, Viral RNA extraction kit, Daejeon, South Korea) according to an already published protocol [37] with modifications [38]. The quality and quantity of the isolated RNA were measured with NanoDrop1000 Version 3.8.1. (Thermo Fisher Scientific).

Reverse transcription from 3 µg of total RNA was performed with the High-Capacity cDNA Archive Kit (Applied Biosystems, Foster City, California, USA) in a total volume of 30 µL according to the manufacturer’s protocol. After dilution with 130 μL of ultrapure water (Applied Biosystems) cDNA was used as template for gene expression analysis. Quantitative real-time PCR (qRT-PCR) was performed on the LightCycler® Nano Instrument (Roche, Basel, Switzerland) using gene-specific primers with SYBR Green protocol as described previously [38]. Briefly: for cycling each 10 μL PCR reaction contained 1 µL cDNA, 250 nM primers and 5 μL FastStart Essential DNA Green Master (2×, Roche). Primers were as follows:

Heat Shock Protein Family A (Hsp70) Member 5 (HSPA5, NM_005347.4), HSPA5-F 5′-AGCCTGGCGACAAGAGTG-3′, HSPA5-R 5′- TCCTTGGGCAGTATTGGATT-3′; the following primer sequences are referenced by [17]: Activating Transcription Factor 4 (ATF4, NM_182810.2, NM_001675.4), X-Box Binding Protein 1 (XBP1, NM_005080.3, NM_001079539.1), DNA Damage Inducible Transcript 3 (DDIT3, NM_004083.5, M_001195056.1, NM_001195055.1, NM_001195054.1, NM_001195053.1), β-actin (ACTB, BC002409.2).

The PCR protocol was as follows: enzyme activation at 95 °C for 10 min, 45 cycles of denaturation at 95 °C for 15 s, annealing at 60 °C and extension at 72 °C for 25 s. All the PCRs were performed with three replicates. After amplification, melting curve was checked to verify the specificity of the PCR reactions. The presented relative gene expression ratios were normalized to ACTB gene, calculated using the comparative CT method (2^−ΔΔCT^). Fold change refers to 2^−ΔΔCt treated^/2^−ΔΔCt untreated^. All values are presented as mean  ±  standard deviation (SD).

### 3.6. Statistical Analysis

Statistical analysis was performed using two-tailed, homoscedastic Student’s t-test to evaluate the statistical significance (set at * *p* < 0.05, ** *p* < 0.01, *** *p* < 0.001) using pairwise comparison of each sample to the untreated control in Microsoft^®^ Excel^™^.

## 4. Conclusions

Despite the emerging field of targeted anti-cancer therapeutics the combination of different therapeutic strategies (surgery, radiation, chemotherapeutic agents, biologics and immunotherapy etc.) may combat cancer and mount an effective therapeutic response [39,40,41,42], however, there is a high need for novel anti-cancer agents with improved cancer cell killing with less side effects.

We designed and synthetized a library of twenty-three achiral curcumin analogs to follow up on our previous work to identify potent, cytotoxic Mannich-base curcuminoids [15,17]. These compounds lack a chiral center so their biological/pharmacological testing is more straightforward without the need for the evaluation of the individual enantiomers. We tested the anti-proliferative activity of our curcumin analogs on human non-small-cell lung carcinoma (A549), hepatocellular carcinoma (HepG2), and pancreatic cancer (PANC-1) cell lines.

**Figure 9:**
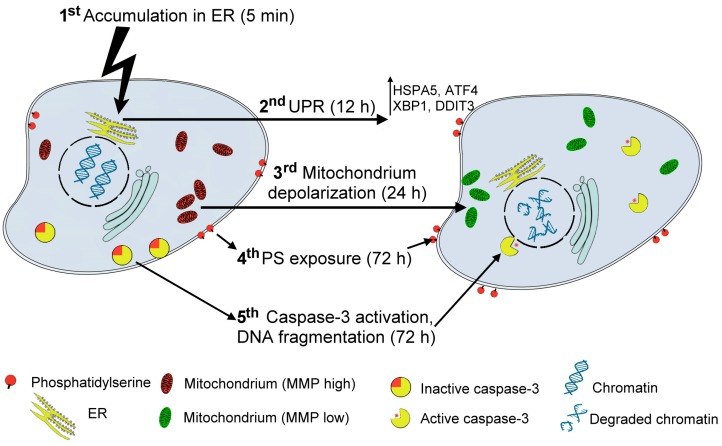
Curcumin and achiral curcumin analogs induce ER stress (1st step) mediated mitochondrial apoptosis in pancreatic PANC-1 cells. The activated unfolded protein response (UPR, 2nd step) induce mitochondrial membrane depolarization (3rd step), PS exposure (4th step) caspase-3 activation and subsequently DNA breakdown (5th step) of pancreatic cancer cells.

Three curcuminoids, C509, C521, and C524 with the highest apoptotic activity were selected for mechanistic studies in the most sensitive PANC-1 cells. We found that the biological activity of the analogs on one hand relied on a cytostatic effect since curcumin and curcuminoid analog treatment led to G_0_/G_1_ cell cycle arrest. On the other hand, our compounds also possessed a potent cytotoxic effect (Figure 9). Their accumulation in the ER caused ER stress, the induction of the unfolded protein response, mitochondrial membrane depolarization, caspase-3 activation, and subsequently DNA breakdown.

Three curcumin analogs, C509, C521, and C524 possessed superior, 40-times more potent cytotoxic activity compared to the natural dihydroxy-dimetoxycurcumin in human pancreatic cancer cells, offering the possibility for their further investigation.

## Figures and Tables

**Table 1:** IC_50_ values (µM) of curcumin (Curc.) and curcumin analogs determined by resazurin assay.

Compound	A549	HepG2	PANC-1
Curc.	27.11	14.53	30.57
C501 ^#^	1.26	0.66	1.44
C502	2.39	>5	2.20
C503 ^#^	1.70	2.55	1.66
C504 ^#^	1.58	1.24	1.40
C505 ^#^	1.29	1.48	1.43
C509 ^#^	2.27	2.98	1.01
C510	>5	1.07	>5
C513	>5	3.11	>5
C514	inactive	inactive	inactive
C515 ^#^	2.73	1.31	1.46
C516	>5	4.19	>5
C517	>5	>5	2.45
C518 ^#^	2.42	4.77	1.25
C519	>5	>5	>5
C520	inactive	inactive	inactive
C521 ^#^	1.88	1.36	1.18
C524 ^#^	1.70	1.69	1.14
C525	inactive	inactive	inactive
C526	inactive	inactive	inactive
C529	inactive	inactive	inactive
C530	inactive	inactive	inactive
C532	>5	>5	1.58
C533	>5	4.81	>5

Cells were treated with curcumin (1.56, 3.125, 6.25, 12.5, 25, and 50 µM) and curcumin analogs (0.16, 0.3125, 0.625, 1.25, 2.5, and 5 µM) in duplicates for 72 h. Viability was examined by resazurin assay as described in Section 3.2. Materials and Methods. Hashmark (^#^) labeled curcumin analogs were selected for further investigation.

**Table 2:** The percentage of apoptotic cells at the lowest applied concentration of 12.5 μM curcumin and 1.25 µM curcumin analogs.

Compound	A549	HepG2	PANC-1
**None**	**2.4** ± 0.44	**2.7** ± 0.37	**1.8** ± 0.02
**Curcumin**	**3.0** ± 0.30	**2.0** ± 0.14	**7.0** ± 0.02
**C501 ^#^**	**1.3** ± 0.06	**2.6** ± 0.55	**6.3** ± 0.42
**C503**	**1.9** ± 0.83	**2.6** ± 0.33	**6.1** ± 0.54
**C504**	**2.0** ± 0.16	**3.4** ± 0.28	**8.8** ± 1.36
**C505**	**1.8** ± 0.11	**5.6** ± 0.31	**8.2** ± 5.83
**C509 ^#^**	**2.7** ± 0.25	**15.4** ± 9.92	**51.0** ± 1.17
**C515**	**3.0** ± 0.77	**3.1** ± 0.11	**22.5** ± 4.43
**C518**	**3.0** ± 0.01	**3.6** ± 0.33	**11.8** ± 2.49
**C521 ^#^**	**2.5** ± 0.5	**8.9** ± 0.13	**37.8** ± 10.86
**C524 ^#^**	**19.8** ± 3.81	**7.3** ± 0.37	**80.4** ± 0.13

Phosphatidylserine exposure was examined as described in Section 3.3.1. Materials and Methods. Analogs labeled with a hashmark (^#^) were selected for further investigation, (± refers to standard deviation)

## Supplementary Materials

Supplementary materials can be found at www.mdpi.com/1422-0067/18/10/2105/s1.

## Abbreviations

AnnV	Annexin V-Alexa Fluor® 488
CDKN2A	Cyclin-dependent kinase inhibitor 2A
CDK4	Cyclin-dependent kinase 4
CDK6	Cyclin-dependent kinase 6
Curc	Curcumin
ER	Endoplasmic reticulum
GLUT1	Glucose transporter-1
GRAS	Generally Recognized as Safe
HCC	Hepatocellular carcinoma
HEPES	4-(2-hydroxyethyl)-1-piperazineethanesulfonic acid
IC50	Half maximal inhibitory concentration
JC-1	5,5′,6,6′-tetrachloro-1,1′,3,3′-tetraethylbenzimidazolocarbocyanine iodide
NSCLC	Non-small cell lung cancer
MAPK	Mitogen-activated protein kinase
MFI	Median fluorescence intensity
MMP	Mitochondrial membrane potential
PBS	Phosphate buffered saline
PI	Propidium iodide
SAR	structure-activity relationship
SD	Standard deviation
UPR	Unfolded protein response
