# Supplementary material for: Achiral Mannich-Base Curcumin Analogs Induce Unfolded Protein Response and Mitochondrial Membrane Depolarization in PANC-1 Cells
Source: Int J Mol Sci. 2017 Oct 7;18(10):2105. doi: 10.3390/ijms18102105 (PMC5666787; doi:10.3390/ijms18102105)
Supplement: Supplementary file 1 [file ijms-18-02105-s001.pdf]

Supplementary materials

Supporting materials and methods

Cell confluency screening

A549 cells (6 x 10^3) were seeded into 96-well plates (Corning Life Sciences) in DMEM/F12 10 % FCS (Gibco). Cells were cultured overnight before treatment. Effects of curcumin and analogs were examined in concentrations presented in the figure after 72h incubation in 100 µl media. We monitored the cell confluency by JuLI™ FL Live Cell Analyser V.0 (NanoEntek) according to the instructions of the manufacturer. We present the confluency determined by the JuLI™ FL Live Cell Analyser after 72h treatment. Data were analyzed by GraphPad Prism® 5.

Supplementary figures:

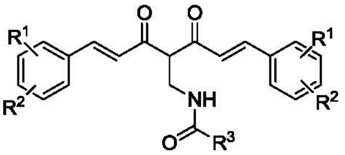

Compound	R^1	R^2	R^3	Molecular weight
**C501**	3-OH		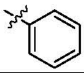	441
**C502**	3-OH		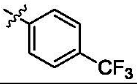	509
**C503**	3-OH		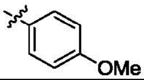	472
**C504**	3-OH		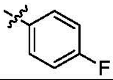	459
**C505**	3-OH		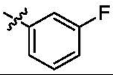	459
**C509**	3-OH		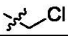	414
**C510**	3-OH		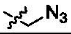	420
**C513**	3-OH		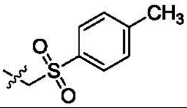	534
**C514**	3-OH		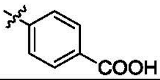	485
**C515**	3-OH		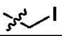	505
**C516**	3-OH		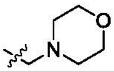	465
**C517**	3-F		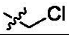	418
**C518**	4-F		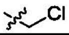	418
**C519**	2-F		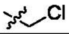	418
**C520**	3-OMe	4-OH	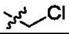	474
**C521**	3-OH	4-OMe	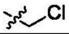	474

**C524**	3-OMe	4-OMe	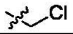	502
**C525**	3-OH	3-OH	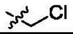	446
**C526**	4-COOH		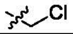	470
**C529**	4-COOH		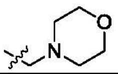	521
**C530**	3-OH	3-OH	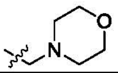	497
**C532**	3-OH		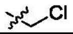	418
**C533**	3-OH		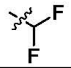	415

Figure S1. Chemical structure of curcumin analogs.

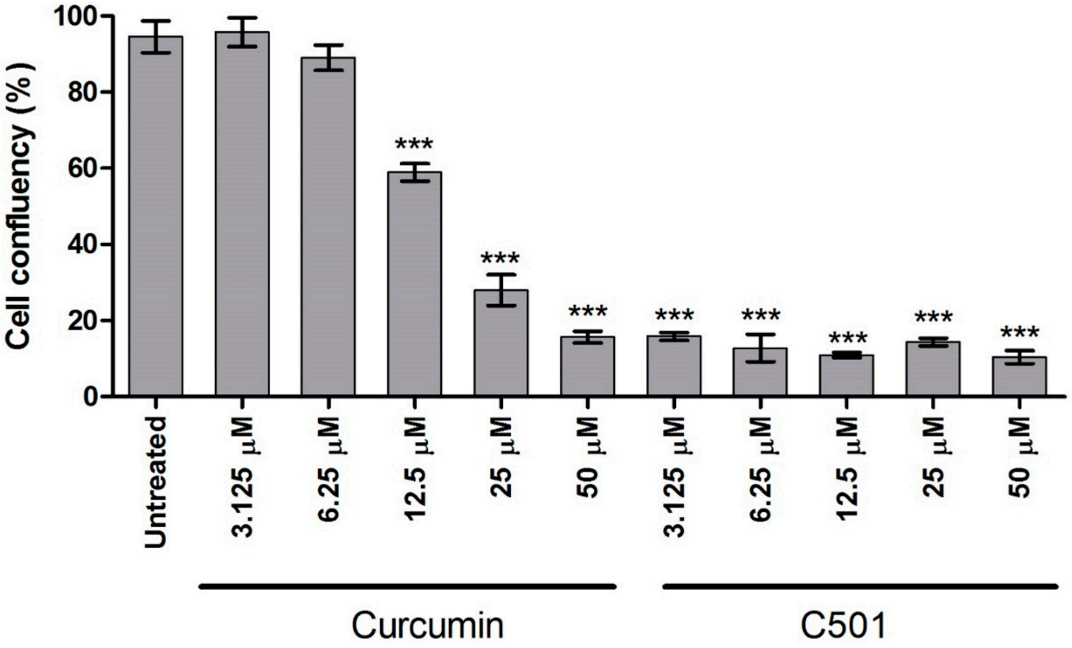

Figure S2. Curcumin and C501 hampered the viability of A549 cells. Cell confluency data of cells treated (from left to right: untreated, 3.125, 6.25, 12.5, 25 and 50 µM) in duplicates for 72h, recorded by Juli™. The arithmetic mean values of two samples ± SD are presented. For experimental details see Supporting materials and methods.

A549 HepG2 PANC-1

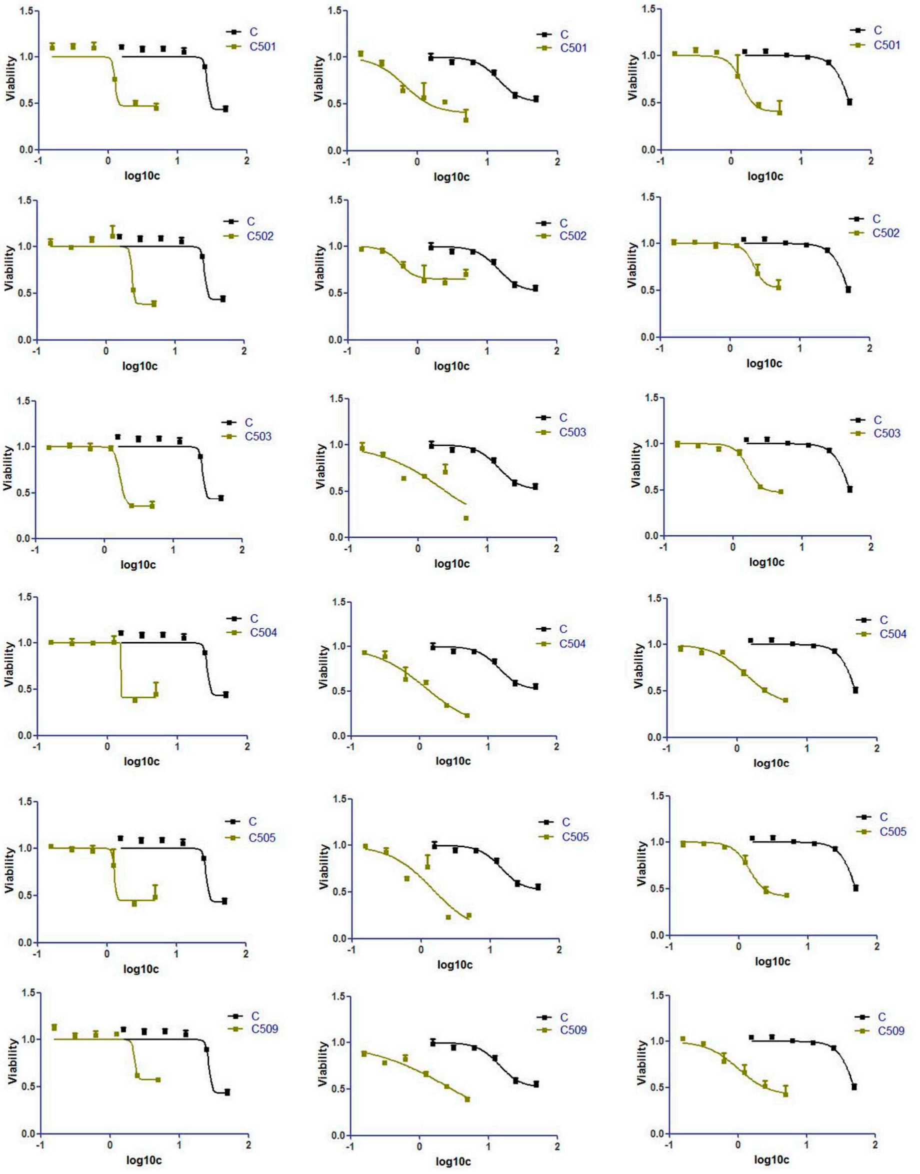

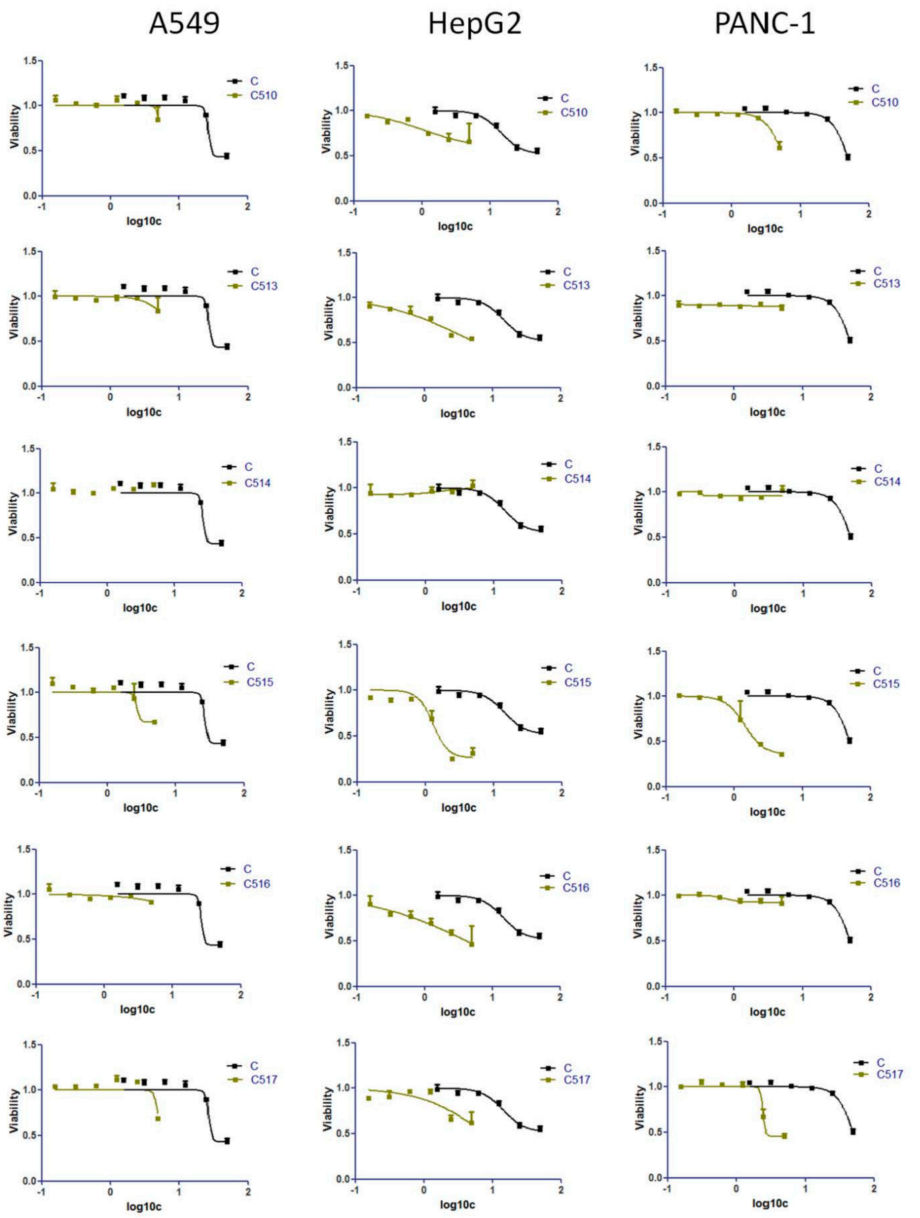
A549
HepG2
PANC-1
Viability
log10c
C
C510
C513
C514
C515
C516
C517

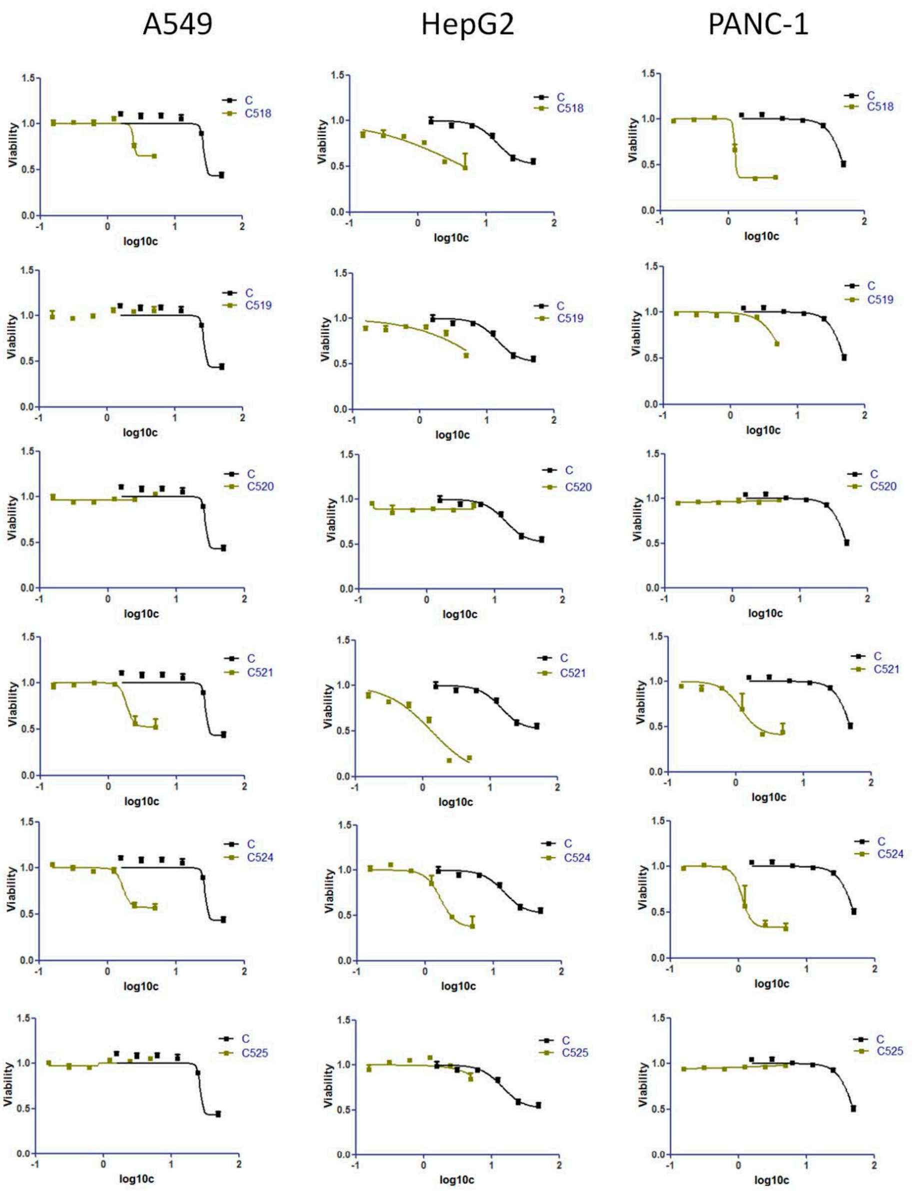
A549
HepG2
PANC-1
Viability
log10c
C
C518
C519
C520
C521
C524
C525

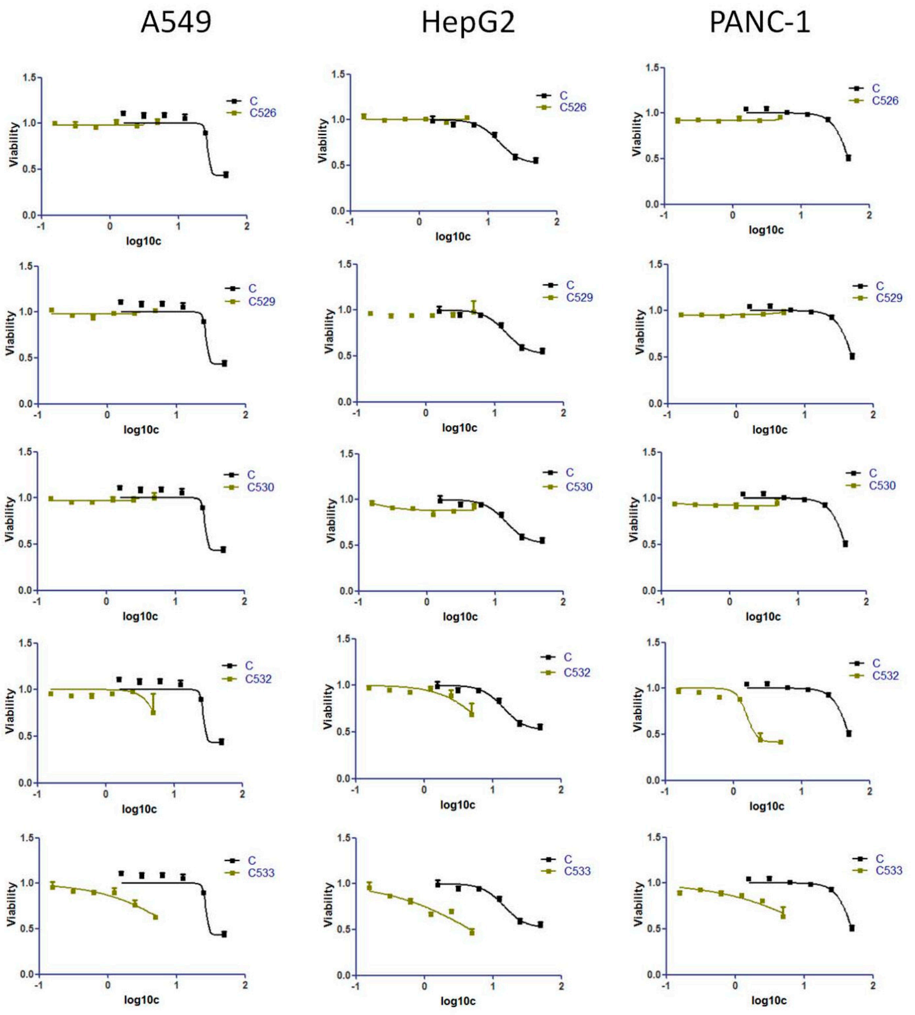

Figure S3. Dose-response curves of curcumin and curcumin analogs on A549, HepG2 and PANC-1 cells. Cells were treated with curcumin (C, 1.56, 3.125, 6.25, 12.5, 25 and 50 μM), and curcumin analogs (0.16, 0.3125, 0.625, 1.25, 2.5 and 5 μM) in duplicates for 72 h. Viability was examined by resazurin assay as described in the 3.2. Materials and methods. The arithmetic means of two samples ± SD are presented.

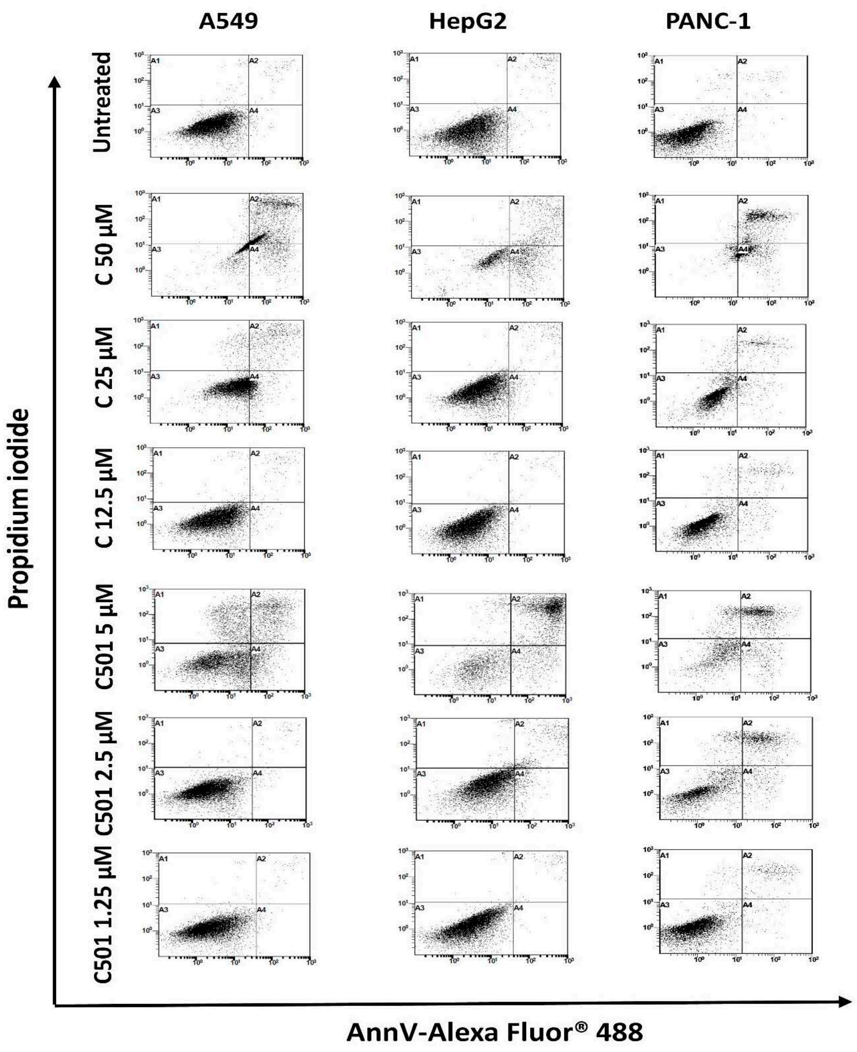
A549
HepG2
PANC-1
Untreated
C 50 µM
C 25 µM
C 12.5 µM
C501 5 µM
C501 2.5 µM
C501 1.25 µM
Propidium iodide
AnnV-Alexa Fluor® 488

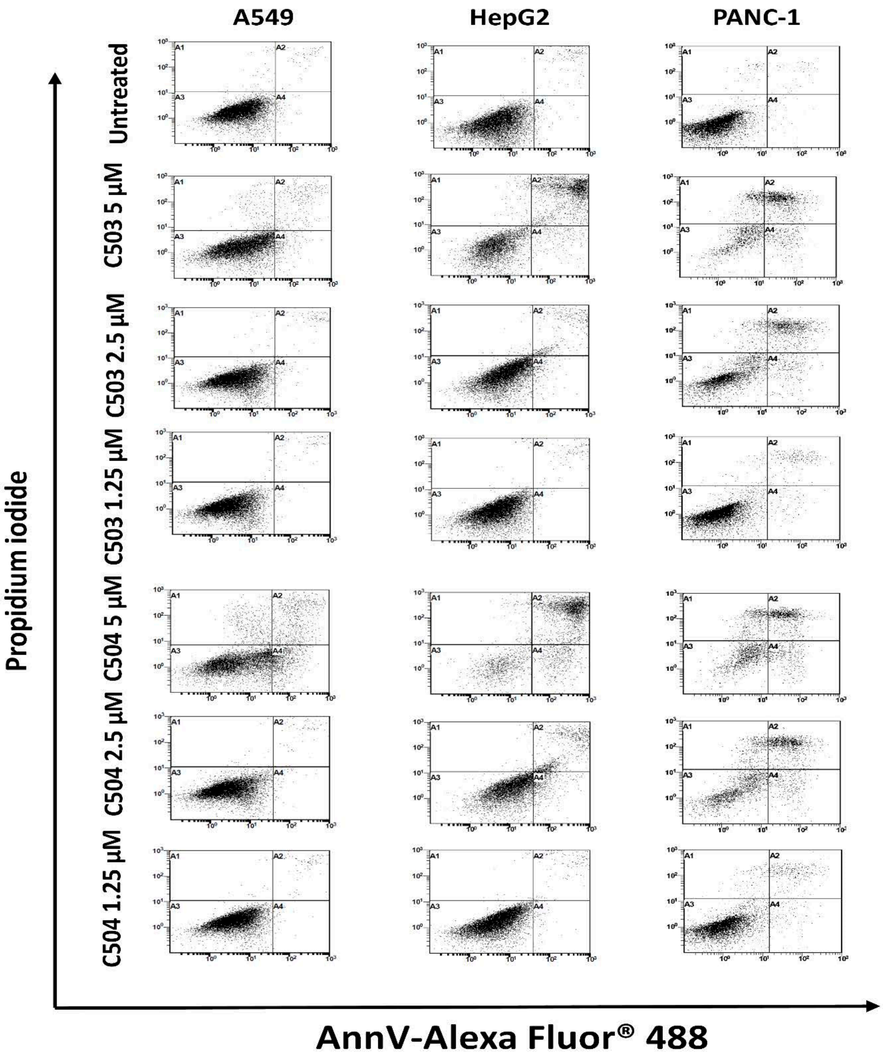
A549
HepG2
PANC-1
Untreated
C503 5 µM
C503 2.5 µM
C503 1.25 µM
C504 5 µM
C504 2.5 µM
C504 1.25 µM
Propidium iodide
AnnV-Alexa Fluor® 488

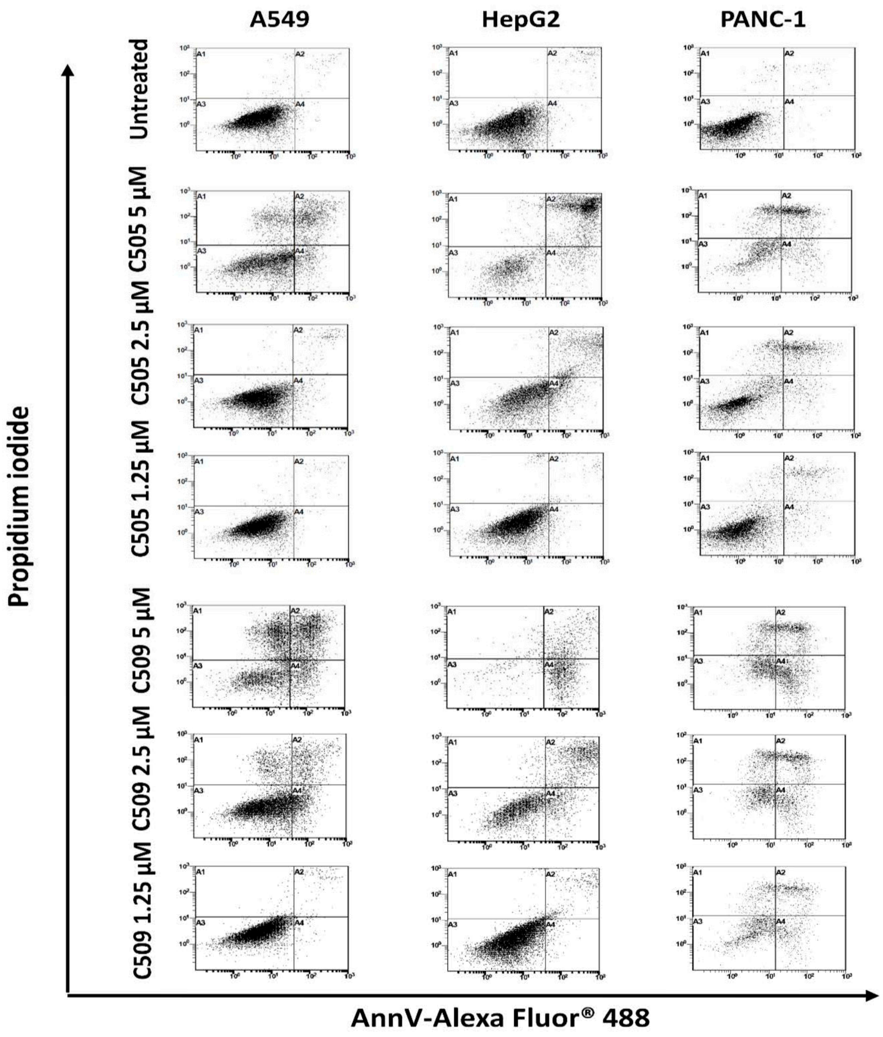

A549
HepG2
PANC-1
Untreated
C505 5 µM
C505 2.5 µM
C505 1.25 µM
C509 5 µM
C509 2.5 µM
C509 1.25 µM
Propidium iodide
AnnV-Alexa Fluor® 488

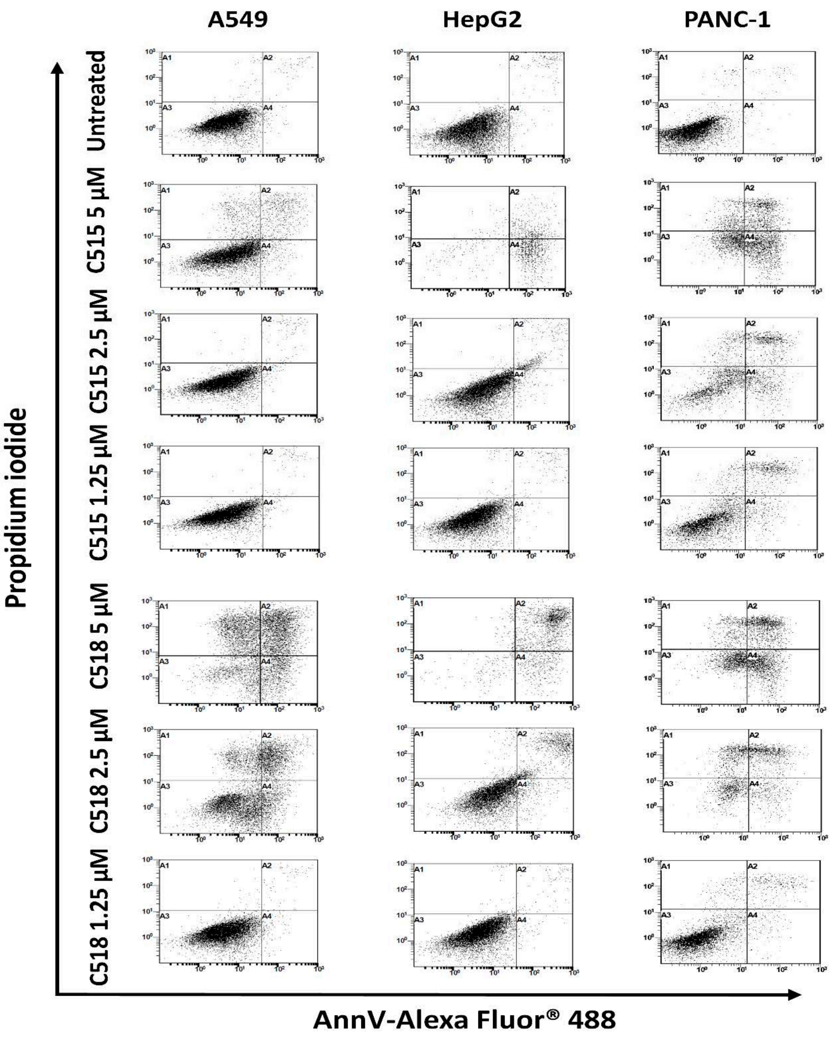

Figure S4. Detection of phosphatidylserine exposure on A549, HepG2 and PANC-1 cells. Representative dot plot images of cells treated with curcumin (C, 12.5, 25 and 50 µM), and curcumin analogs (1.25, 2.5, 5 µM) in duplicates for 72 h. Phosphatidylserine exposure was detected as described in 3.3.1. Materials and methods.

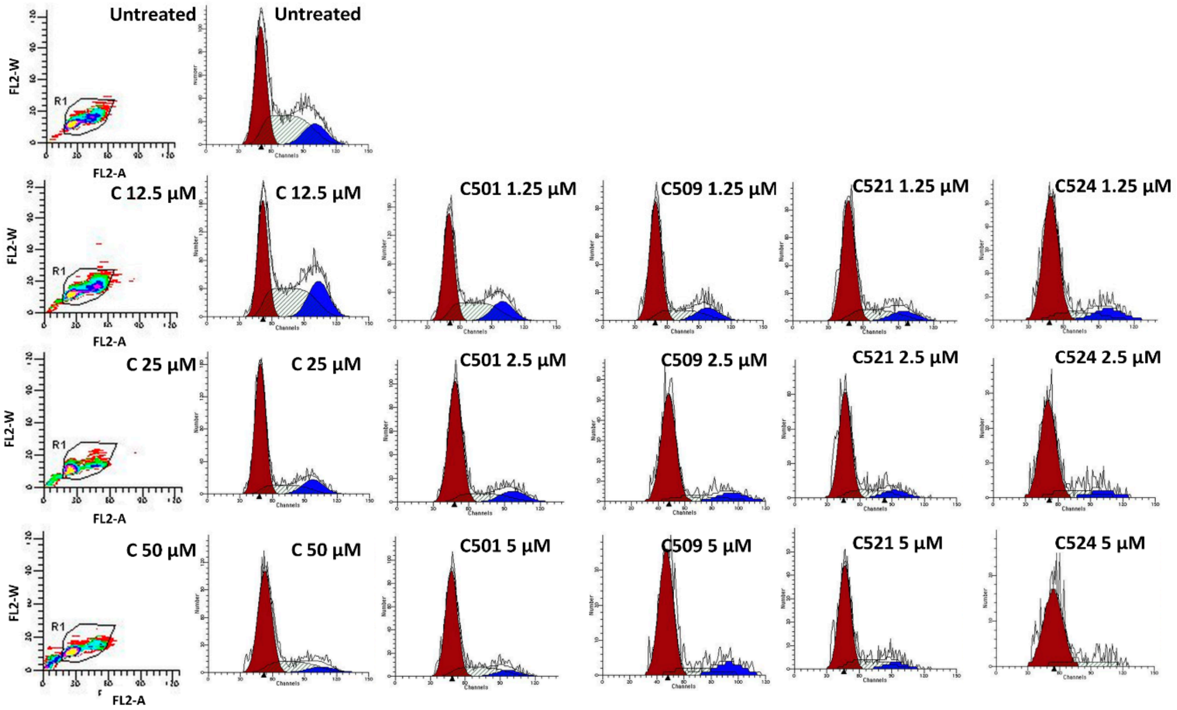

Figure S5. Cell cycle analysis of PANC-1 cells upon curcumin and curcumin analog treatment. Cells were treated with curcumin (C, 12.5, 25 and 50 μM) and curcumin analogs (1.25, 2.5 and 5 μM) in duplicate for 72 h. Representative images show cell cycle distribution analysis as described in the 3.3.2. Materials and methods.
